# Case Report of Three Immature Cystic Teratomas in Northern Ghana

**DOI:** 10.1155/2019/1210509

**Published:** 2019-03-14

**Authors:** E. M. Der, S. Seidu

**Affiliations:** ^1^Department of Pathology, School of Medicine and Health Science of the University for Development Studies and the Tamale Teaching Hospital, Ghana; ^2^The Preysterian Hospital, Bawku, Upper East Region of Ghana, Ghana

## Abstract

**Background:**

Preoperative diagnosis of immature cystic teratoma can be challenging for clinicians. In this report, we present three cases.

**Methods:**

We describe three women aged 10, 20, and 23 years, respectively, who presented with abdominal masses which were diagnosed by abdominal ultrasound as mature cystic teratomas. All women had emergency laparotomy and oophorectomy.

**Results:**

Histopathological examination reported these ovarian tumours to be immature cystic teratomas. This case report also provided a brief summary of the clinicopathological features of all ovarian teratomas diagnosed in two centres during the period of review.

**Conclusion:**

Immature ovarian teratoma affects primarily younger patients; it is important for clinicians to have a high sense of suspicion whenever the diagnosis of a germ cell tumour is entertained.

## 1. Introduction

Ovarian teratomas are the most common germ cell neoplasms and the reasons for which most oophorectomies are performed [1, 2]. A number of histological subtypes have been documented, all of which contain mature or immature tissues of germ cell origin. The most common of these tumours, the mature cystic teratomas (MCT), typically contain mature tissues of ectodermal (skin, brain), mesodermal (muscle, fat), and endodermal (mucinous or ciliated epithelium) origin [2, 3]. In monodermal teratomas, one of these tissue types predominates. For instance, in struma ovarii, thyroid tissue dominates.

Like mature cystic teratomas, immature cystic teratomas (IMCT) of the ovary are composed of immature tissues derived from the three germ layers. IMCTs are typically larger (14–25 cm) than mature cystic teratomas (average, 7 cm) [4–6]. These neoplastic lesions differ from mature cystic teratomas in that they demonstrate clinically malignant behaviour, are much less common (<1% of ovarian teratomas), and affect the younger age group (usually during the first 2 decades of life) with peak occurrence being between 15 and 19 years of age [4, 7–10]. Immature teratomas rarely appear during the menopausal period [10].

IMCTs are histologically distinguished by the presence of immature or embryonic tissues [4, 7]. They may be solid or have a prominent solid component with cystic elements [7]. The cystic areas are usually filled with serous or mucinous fluid or may be filled with fatty sebaceous material.

Although the typical histopathological features of MCT of the ovary are well described, the less common types of ovarian teratomas, particularly the immature teratomas, have received less documentation in Ghana. This case report presents three Ghanaian women with abdominal masses that were diagnosed clinically as mature cystic teratomas; however, histopathological examination reported these masses as immature teratomas. This case report also provides brief data on the clinicopathological features of all ovarian teratomas, including the immature forms, diagnosed in two centres during the period of review.

## 2. Case Report

### 2.1. Case One

A 23-year-old young woman presented at the Obstetrics and Gynaecology Department of the Tamale Teaching Hospital (THH) with abdominal swelling of four months duration and a month's history of abdominal pain. Physical examination revealed a right-sided tender mass, mobile in all the planes. A pelvic ultrasound diagnosis of a benign ovarian mass with cystic components was made. All the other systems were essentially normal. She had emergency laparotomy and right oophorectomy performed. The specimen was sent to Der Medical Diagnosis Centre for histopathological examination.

#### 2.1.1. Histopathology

**Figure 1:**
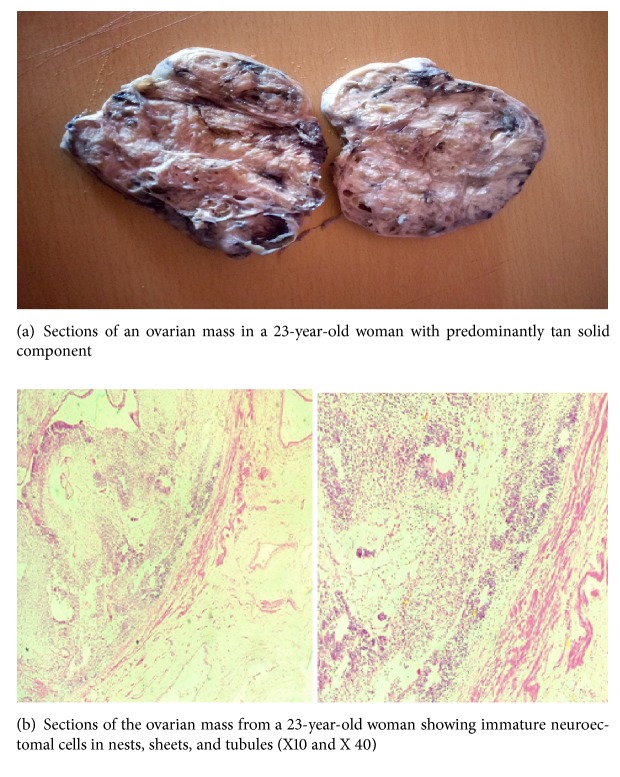
Case one: a 23-year-old woman with right ovarian mass.

*Gross*. A tan nodular mass measuring 12.5x10.0x8.5cm was received in the diagnostic centre. Slicing through the mass revealed a lesion with predominantly tan to whitish grey solid component with few cystic cavities (Figure 1(a)). 

*Microscopic Examination*. Sections of representative portions of the ovarian mass showed predominantly a solid lesion with few cystic areas. The cysts were lined by squamous, gastrointestinal, and respiratory type epithelium. The solid component was composed largely of islands, nests, sheets, and tubules of immature neuroepithelial cells or elements. There were islands of mature brain tissue, adipose tissue, cartilage, and skeletal muscles in the stroma (Figure 1(b)). 

*Histological Diagnosis*. Right ovary (oophorectomy): immature cystic teratoma.

### 2.2. Case Two

A 10-year-old girl presented at the outpatient department of the Upper East Regional Hospital, Bolgatanga, with a six-month history of recurrent intra-abdominal pain. Examination revealed a painful mobile intra-abdominal mass. Abdominopelvic ultrasound conducted suggested a mature cystic teratoma. She was referred to the Obstetrics and Gynaecology Unit of the same hospital where she had an emergency laparotomy carried out. The specimen was sent to Der Medical Diagnostic Centre in Tamale, in the Northern Region for histopathological examination.

#### 2.2.1. Histopathology

**Figure 2:**
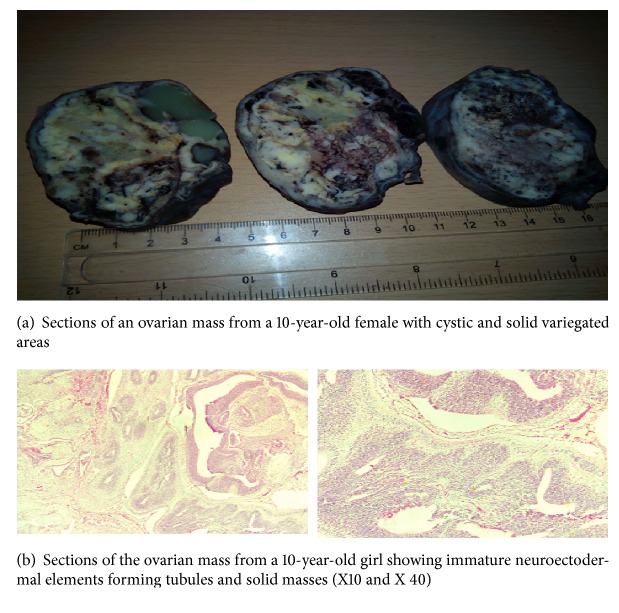
Case two: A 10-year-old girl with right ovarian mass.

*Gross*. A tan nodular mass measuring 19.0x17.0x7.5cm was received at the Diagnostic Centre. The cut surface was variegated with solid and cystic components. The cysts were filled with cream-coloured semisolid material (Figure 2(a)). 

*Microscopy*. Sections of representative portions of the ovarian mass showed a multicystic lesion with solid components. The cysts were lined by mature and immature ectodermal elements. The solid components consisted predominantly of sheets, nests, and tubules of immature neuroepithelial cells or elements. The immature mesenchymal elements were cartilage, bone, skeletal muscle, and ocular elements (Figure 2(b)). 

*Histological Diagnosis*. Right ovary (oophorectomy): immature cystic teratoma.

### 2.3. Case Three

A 20-year-old adolescent girl presented at the outpatient unit of the Presbyterian Hospital in Bawku in the Upper East Region of Ghana with a left intra-abdominal mass of more than five-month duration. A diagnosis of a mature left ovarian cystic teratoma was made based on the ultrasonographic findings. She had laparotomy and oophorectomy and the specimen was sent to Der Medical Diagnostic Centre in Tamale for histopathological examination.

#### 2.3.1. Histopathology

*Gross*. An ovarian mass that measured 17.0x14.0x8.0cm was received. The cut surface of the mass was multicystic with tan nodular solid components. The cysts were filled with cream-coloured semisolid material. 

**Figure 3:**
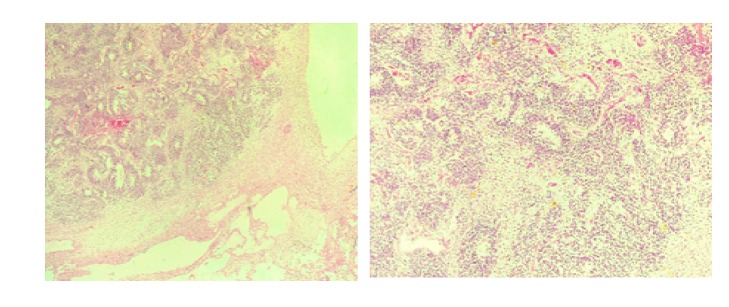
Case three: A 20-year-old woman with left ovarian mass. Sections of the ovarian mass from a 20-year-old woman showing immature neuroectodermal elements forming tubules and solid masses (X10 and X 40).

*Microscopic Examination*. Sections of representative portions of the ovarian mass showed a lesion with cystic and solid components. The cysts were lined by squamous and respiratory type epithelium. The solid component was composed predominantly of immature neuroepithelial cells or elements in sheets and tubules. There were areas of haemorrhages and necrosis (Figure 3). 

*Histological Diagnosis*. Left ovarian mass (oophorectomy): immature cystic teratoma.

## 3. Discussion

Immature ovarian cystic teratomas (IMCT) are a less common subtype of ovarian teratomas with malignant clinical behaviour; they are histologically distinguished by the presence of immature or embryonic tissues, particularly neuroectodermal elements [4–10]. The three immature cystic teratomas described above form part of a total of 32 ovarian teratomas diagnosed in two centres in the tamale metropolis during the period July 2012 to July 2018, namely, pathology department of TTH and Der Medical Diagnostics Centre. Thus, IMCTs represent 9.4% of our cases of germ cell tumours. This is much higher than the 1.4% prevalence rate reported in the study by Akpakpo et al. [11] of the pathological and clinical features of 706 primary tumours of the ovary in the largest tertiary hospital in Ghana. The difference here is that the sample size in the current case report was 32 with 3 cases of IMCTs over a 7-year period; however, that of Akpakpo et al. was a histopathological review over a 10-year period with sample size of 706 with only 4 cases of IMCT.

All the females diagnosed with IMCT in this current study were within the second and third decades of life. This is in line with previous reports that IMCT affects the younger age group, usually during the first two decades of life with a peak occurrence between 15 and 19 years of age, [4, 8–10] and that the disease rarely appears during the menopausal period [10, 11].

All the women presented clinically with abdominal masses were associated with some degree of pain. These symptoms are not specific to IMCT but may manifest as a result of any intra-abdominal neoplastic condition. This is further illustrated by the fact that all were preoperatively diagnosed with the aid of an imaging technique as mature cystic teratomas. The nonspecific clinical presentation of IMCT and the difficulties in making a definitive clinical diagnosis has been expressed by previous studies on ovarian tumours [11, 12]. Similar to our experience, there are other reported cases with pathological results of immature teratoma where the initial diagnosis was missed because the preoperative imaging technique could not be differential between mature and immature neural elements [1, 8]. For instance, Yamaoka et al. [13] reported that the radiographic findings of immature cystic teratomas are similar to those of mature cystic teratomas and hence this poses a challenge in arriving at a clinical diagnosis.

The ovarian masses in our study were generally large, the smallest being 12.5x10.0x8.5cm and the largest 19.0x11.0x7.5cm. Large ovarian masses, especially in younger females, must be carefully sampled during macroscopic examination of the specimen by the pathologist and examined histologically as illustrated in these cases for evidence of immature ectodermal, endodermal, and mesodermal elements, the presence of any of which defines immature teratoma which has the potential of distant spread. This is so because IMCTs have been reported to be associated typically with bigger ovarian size compared to the mature cystic teratomas [5, 6, 12, 13]. The duration of presentation for our cases ranged from 4 to 6 months from the clinical history provided and this was similar to that reported in the study by Akakpo et at. [11] in Accra, Ghana.

## 4. Conclusion

The current case reports illustrate the difficulties encountered by clinicians in making a diagnosis of ovarian neoplasms based on clinical history and imaging techniques alone. It is recommended that, in making a diagnosis of ovarian neoplasm especially in younger females, immature cystic teratoma is considered as a differential diagnosis and that the pathologist should have a high index of suspicion and therefore carefully sample and examine the mass histologically for evidence of immature elements that are diagnostic of immature teratoma.
